# Physical activity in Iranian older adults who experienced fall during the past 12 months

**DOI:** 10.1186/1471-2318-14-115

**Published:** 2014-10-31

**Authors:** Leili Salehi, Behjat Shokrvash, Ensiyeh Jamshidi, Ali Montazeri

**Affiliations:** Department of Health Education and Promotion, School of Public Health, Alborz University of Medical Sciences, Karaj, Iran; Department of Health Education and Promotion, School of Public Health, Tabriz University of Medical Sciences, Tabriz, Iran; Department of Health Education and Promotion, School of Public Health, Tehran University of Medical Sciences, Tehran, Iran; Community Based Participatory Research Center, Iranian Institute for Reduction of High-Risk Behaviors, Tehran University of Medical Sciences, Tehran, Iran; Mental Health Research Group, Health Metrics Research Center, Iranian Institute for Health Sciences Research, ACECR, Tehran, Iran; Faculty of Humanity Sciences, University of Science & Culture, ACECR, Tehran, Iran

## Abstract

**Background:**

Physical activity may have several benefits for elderly people. However, the risk of falling might prevent this population from showing interest in physical activity. This research was aimed to explore facilitators and barriers to physical activity in older persons who have experienced at least one fall in the past 12 months.

**Methods:**

This cross sectional study was conducted in 2010-2011, in Tehran, Iran. Using a multistage sampling method a group of elderly people entered into the study. A multi-section questionnaire was used to collect data on demographic information, physical activity level, and different determinants that might influence physical activity. Several statistical tests including linear regression were used to analyze the data.

**Results:**

In all, 180 old people from 40 elderly centers (49 men and 131 women) took part in the study. The mean age of participants was 65.9 ± 6.1 years. The result indicated that most participants experienced two or more falls during the last year (54.5%). Those who had more falls significantly scored lower on the Physical Activity Scale for Elderly (p < 0.0001). ‘Keeping in touch with friends’ was the most important advantage cited by participants for performing physical activity. The results obtained from linear regression analysis showed that ‘perceived benefits’ was the only significant factor that associated with physical activity (β = 1.03, p < 0.001).

**Conclusion:**

The findings suggest that perceived benefits could facilitate physical activity among elderly regardless of number of falls, self-reported health and daily living activities. However, we observed inverse association between number of falls and physical activity. Indeed the findings suggest that we should reinforce benefits exist when designing programs to increase physical activity among elderly population.

## Background

Fall is one of the main contributing factors for accidental deaths and the foremost cause of injury admissions to acute-care hospitals in adults older than 65 years of age [1]. It has been estimated that more than one-third of adults over the age of 65 years fall annually [2]. The majority of fractures among older adults are due to falls [3]. Of those who experience a fall, hip fracture [4] and other injuries are common [5]. Falling might lead to fear of subsequent falls [6].

Consistent with the global trend of aging, the proportion of the older population in Iran has also increased over the last few decades [7]. According to the United Nations Statistics, proportion of the older in Iran was 5.4 percent in 1975 and will be increased by 10.5% in 2025 and by 21.7% in 2050 [8]. This dramatic demographic change will inevitably lead to a significant economic impact on the Iranian health care system. The aging population, with its high prevalence of falls, will incur greater health care service costs.

The risk of falls in the elderly might be influenced by several lifestyle factors such as physical activity (PA) [9]. However, despite existing evidence for advantages of physical activity for successful aging [10], the majority of the Iranians elderly do not engage in regular PA [11]. Approximately 30% of older adults who have experienced a fall reported that they have a fear of falling [12] and the fear of falling was found to be high in elderly who had fallen during the previous year [13]. Curcio et al. found that these individuals may experience a greater decrease in activity level and functional status, weak perceived health and worse depressive symptoms [14]. Also an increased risk of falls is linked to decreased mobility as a result of physical inactivity and ageing [15]. However although many researchers from Iran and other countries have explored the perceptions of older adults towards exercise and fitness [11, 16, 17], few studies aimed to explore perceptions of older adults who experienced falls [18]. This research is aimed to explore facilitators and barriers to PA in older persons who have experienced at least one fall in the past 12 months.

## Methods

### Design and data collection

This population-based cross-sectional survey was conducted between October 2011 and June 2012 to investigate physical activity and its related factors in elderly who experienced at least one fall during the past 12 months. Physical activity was defined as ‘any bodily movement produced by skeletal muscles that requires energy expenditure’. As suggested by the World Health Organization physical activity includes exercise as well as other activities which involve bodily movement and are done as part of playing, working, active transportation, house chores and recreational activities [19]. To collect data, trained interviewers conducted face-to-face interviews with a random sample of elderly people in Tehran, Iran.

### Sampling

The study sample was recruited from the elderly centers in Tehran, Iran. There are 40 elderly centers located in different geographical areas in Tehran. The centers are affiliated to municipality offering free services to old adults who become a member. Both male and females could become a member. The only criterion for membership is that the members should have at least 60 years old. A multistage sampling method was applied to recruit the study sample. First Tehran was divided into north, south, west and east. Then within each geographical location proportional to the number of centers the units of sampling were identified. Finally within each unit (elderly centers) from the list of members individuals were invited for the study. The individuals who did not agree to take part in the study, those who had a history of hospitalization or were suffering from serious illness or experienced surgery (up to 3 months before the study commence) were excluded.

### Instruments

A multi-section questionnaire was developed to assess physical activity among Iranian elderly. The questionnaire consisted of the following sections:

1. *Demographic and anthropometrics characteristics:* included recording of demographic and anthropometric data such as age, gender, education, marital status, weight, height, living arrangement, chronic disease and number of falls during the last year.
2. *Physical Activity Scale for Elderly (PASE)*: The modified version of PASE was used to measure self-reported physical activity. It is a brief and easily scored questionnaire designed specifically to assess PA over a week time in epidemiologic studies of elderly [20]. The questionnaire contains three sections regarding the frequency and duration of leisure-time activities, household activities, and work-related activities. The overall PASE score ranges from 0 to 400 or more.
3. *Perceived benefits and barriers regarding physical activity:* The third part of the questionnaire was about different determinants that might influence physical activity including facilitators and barriers to physical activity. We developed a 16-item questionnaire for the advantage and disadvantage of physical activity. In fact the questionnaire was derived from the Decision Balance Scale [21] and two focus group discussions with 20 elderly Iranians who experienced at least one fall during the last year (10 people in each group). We asked participants to discuss about pros and cons of physical activity considering their own and others’ experiences. In fact they were prompted to think about benefits of and barriers to physical activity for themselves and for others. Then benefits and barriers generated by participants in addition to what we derived from the Decision Balance Scale were reviewed and written in item form. All items were reviewed and edited for clarity and conciseness. Finally we developed a questionnaire that contained two subscales: the Benefits of PA subscale and the Barriers to PA subscale. The Benefits of PA subscale consisted of 10 items with a 5-point Likert response categories ranging from ‘not at all important’ to ‘extremely important’. An additive score was constructed to provide an overall estimate of the perceived benefits of physical activity, with possible score ranging from 10 to 50 points. The Barriers to PA subscale consisted of six items with a 5-point Likert response categories ranging from ‘not at all important’ to ‘extremely important’ with possible score ranging from 6 to 30 points. The Cronbach’s alpha coefficient for both subscales was 0.8, well above the recommended threshold for internal consistency of a questionnaire.
4. *Additional measures:* (I) we used a single item asking respondents to rate their present health on a 5-point Likert scale ranging from 1 = poor to excellent = 5. (II) Activity of Daily Living (ADL) also was assessed by a structured interview using a 12-item questionnaire including items on mobility and walking, hand manipulation, balance, and changing posture with a 4-point Likert scale response categories ranging from ‘completely unable to do’ to ‘without any help’ with possible score ranging from 12 to 36 points. The Japanese Ministry of Education, Culture, Sports, Science and Technology had originally introduced the ADL questionnaire for the assessment of the elderly, with some modifications [22].

### Data analysis

Descriptive analysis was used to explore the data. In addition different statistical tests including t-test, one-way analysis of variance, chi square, and multiple linear regression analysis were used for comparisons and assessing the association between dependent (physical activity) and independent variables. The independent variables entered into the regression models were: perceived barriers, activities of daily living and perceived benefits, self reported health and number of falls. The inclusion of these variables in the model was due the fact that perceived benefits and perceived barriers are the main components of the health belief model. It is believed that this model is one of the most appropriate models that could explain and predict health-related behaviors [23].

### Ethics

Ethic committee of Tehran University of Medical Sciences approved this study. Before conducting any procedure, the aim, method and confidentiality of the study were explained completely to the potential participants and if they agreed to participate in the study, they were asked to read and sign the consent form.

## Results

Overall, 180 old people from 40 elderly centers (49 men and 131 women) took part in the study. The mean age of participants was 65.89 (SD = 6.1) years ranging from 60 to 85. The majority of participants were married (53.2%), suffering from chronic diseases (78.8%), and experienced two or more falls during the last year (54.5%). The mean PASE score for participants was 56.2 ± 46.7. Those who had more falls significantly scored lower on the PASE (p < 0.0001). The characteristics of study sample and the mean PASE scores by sub-groups are shown in Table 1.

**Table 1:** **Mean scores of the PASE by demographic characteristics of the study sample (n** = **180)**

	No (%)	PASE score (95%CI)	P value*
**Age**			0.004
60-64	88 (48.8)	68.0 ± 55.2 (56.3-79.7)	
65-69	44 (24.4)	57.6 ± 37.6 (46.2-69.1)	
70-74	26 (14.4)	33.9 ± 24.4 (24.1-43.8)	
75-79	11 (6.1)	22.5 ± 11.0 (15.1-29.9)	
>80	11 (6.1)	42.2 ± 29.0 (22.6-61.8)	
**Gender**			0.725
Male	49 (27.2)	58.2 ± 44.8 (45.3-44.8)	
Female	131 (72.7)	55.4 ± 47.5 (47.2-63.7)	
**Education**			0.426
Illiterate	102 (56.6)	58.6 ± 48.0 (49.2-68.1)	
Literate	78 (43.3)	53.0 ± 45.0 (42.9-63.2)	
**Marital status**			0.703
Married	100 (55.5)	54.8 ± 42.7 (46.3-63.3)	
Never married	2 (1.1)	22.9 ± 7.1 (41.1-87/0)	
Divorced	7 (3.8)	63.6 ± 25.1 (40.3-86.9)	
Widowed	71 (39.4)	53.3 ± 53.8 (45.6-71.1)	
**Having chronic disease**			0.148
Yes	142 (78.8)	53.1 ± 46.4 (45.3-60.8)	
No	38 (21.1)	65.9 ± 45.4 (51.0-80.8)	
**Living arrangement**			<0.001
Living with others	164 (91.1)	57.3 ± 48.3(49.9-64.8)	
Living alone	16 (8.9)	44.8 ± 22.1(33.0-56.6)	
**Number of falls**			<0.001
1	82 (45.5)	87.6 ± 51.5(76.2-98.9)	
2	55 (30.6)	39.24 ± 15.8(34.9-43.5)	
3	21 (11.7)	22.98 ± 11.3(17.8-28.1)	
4	22 (12.2)	13.7 ± 4.3(11.8-15.6)	

*Derived from t-test or one-way analysis of variance (where necessary) for comparing PASE score among different subgroups.

### Perceived benefits and barriers

Participants cited ‘keeping in touch with friends’ (n = 73, 40.5%) as the most important advantage of physical activity followed by ‘it is fun’ (n = 65, 36.1%) and ‘would live longer’ (n = 55, 30.5). Regarding barriers, the findings revealed that ‘sickness’ was the most frequently reported barrier to physical activity (n = 81, 45.0%) followed by ‘fear of falling’ (n = 67, 37.2%). The detailed results are shown in Table 2.

**Table 2:** **Frequency of responses to survey questions regarding the benefits and barriers of physical activity**

Perceived benefits		No.	%
	Physical activity improves my mental health	19	10.6
	Physical activity make me feel better in general	29	16.1
	Physical activity improve my balance	24	13.3
	Physical activity improve my flexibility	26	14.4
	Physical activity reduce risk of falling	17	9.4
	Physical activity helps me sleep better at night	24	13.3
	I will live longer if I were physically active	55	30.6
	I have a more positive outlook on life with physical activity	27	15
	Physical activity lets me keep in contact with friends	73	40.6
	Physical activity is fun for me	65	36.1
**Perceived barriers**			
	I fear of falling	67	37.2
	I am too sick to be physically active	81	45.0
	I have lack of motivation	51	28.3
	I am too exhausted to do physical activity	47	26.1
	I don’t have anyone to do physical activity with	67	37.2
	I don’t have secure environment to be physically active	67	37.2

### Predicting factor

The results obtained from the linear regression model indicated that perceived benefits was the only significant factor that predicted physical activity among the study participants regardless of number of falls and other independent variables studied (β = 1.03 P < 0.001). However, we observed inverse association between number of falls and physical activity. The results are shown in Table 3.

**Table 3:** **Factors predicting physical activity in elderly who experienced fall during past 12 months**

Determinants	Mean (SD)	Un standardized coefficients		Standardized coefficient	t	P
		β	SE	β		
**Age**	65.89 (6.1)	−0.11	0.27	−0.02	-.0.415	0.679
**Living arrangement (living with someone vs. living alone)**	NA	0.894	4.76	0.005	0.188	0.851
Perceived benefits*	20.6 (8.6)	5.59	0.32	1.03	17.33	<0.001
Perceived barriers**	17.2 (5.0)	−0.462	.28	−0.05	−1.62	0.105
Self reported health***	3.4 (1.43)	3.54	2.10	0.11	1.68	0.094
ADL****	12.1 (3.4)	0.104	0.49	0.01	0.210	0.834
**Number of falls**	1.9 (1.1)	−2.22	2.01	−0.05	−1.10	0.271

*Score ranged from 10 to 50 with higher scores indicating more perceived benefits.

**Score ranged from 5 to 30 with higher scores indicating more perceived barriers.

***Score ranged from 1 to 5 with higher scores indicating a better self reported health.

****Score ranged from 12 to 36 with higher scores indicating better conditions.

## Discussion

This study specifically focused on elderly people who had experienced at least one fall during the last year. The findings showed that the participants had low levels of physical activity and this was almost fifty percent lower than for elderly Iranians who had not fallen [11]. There is a possibility that this population has unique facilitators and barriers to participate in physical activity. Therefore this study aimed to determine several factors that may influence PA level in the elderly.

‘Keeping in touch with friends’ was mentioned as the primary reason for engaging in physical activity. This result was consistent with another study reported from Iran [11]. ‘To have fun’ was the second important factor that was cited by 36.1% of the elderly. Fun or enjoyment was one of the most motivating and encouraging reasons cited in the literature for most age groups to be engaged in physical activity [24].

‘I am too sick to do physical activity’ was also mentioned as a top reason for lack of participation in PA in this study. Another study also revealed physical illness as a barrier to physical activity [25]. It appears that for fall prevention strategies to be effective, exercise educational programs should not be undertaken in isolation, but in partnership with other interventions and integrated into generic health.

Fear of falling was identified as the second top barrier in the current study. Another study showed that up to 70% of recent fallers acknowledge fear of falling [26]. Suzuki [27] reported that 85% of older adults with a history of fall had fear of falling and 34% said that they did not go out because of the anxiety of fall again. Similarly we found that there was a significant relationship between number of falls and the PASE score. The association between fear of falling and PA was also found in another study where of the 54 elderly who reported fear of falling, 41 subjects (75.9%) reported activity modification secondary to fear [28].

‘I don’t have anyone to do physical activity with’ was among top barriers (37.2%) that acknowledged by participants. Similarly a study on sedentary behaviors among elderly Mexican and European Americans also found that companionship was a predominant barrier to physical activity [29].

The current study showed no significant differences regarding physical activity between males and females. However studies from other countries indicated contrary results [30]. This difference might be explained by the existing differences in the life styles of Iranian women. Indeed, in the context of Iranian living conditions, elderly women have high levels of physical activity due to their daily duties such as household activities, and caring for grandchildren.

The results obtained from this study were also consistent with other studies [31] where perceived benefits were predictors of physical activity. However, one should be aware that there are several factors that might affect physical activity among older population. Thus interventions aimed at changing multiple risk factors can be more effective than those targeting one risk factor alone.

## Limitations

Given that all our respondents were members of elderly centers, the findings of this study might not be generalized to all elderly who live in Tehran. These elderly might differed from others in terms of family cohesiveness, and social support. We did not collect data for these, but given that most participants were married and living with someone, we speculate that our sample had better conditions than the general older population. In addition since we could not find well-validated Iranian version of instruments we needed for this study, most of the information has been collected through questionnaires developed by the authors. Thus, further studies are needed for evaluation of lifestyle in a larger and more diverse group of elderly using well-validated measures.

## Conclusion

The results of this study indicated that perceived benefits was the most significant predicting factor for physical activity among elderly regardless of number of falls, self-reported health and daily living activities. However, we observed inverse association between number of falls and physical activity. Indeed the findings suggest that we should reinforce benefits exist when designing programs to increase physical activity among elderly population.

## Abbreviations

PA: Physical activity
PASE: Physical activity scale for elderly
ADL: Activity of daily living.

## References

[1] Peel NM (2011). Epidemiology of falls in older age. Can J Aging.

[2] Centers for Disease Control and Prevention (CDC): **Falls among older adults: an overview.** http://www.cdc.gov/HomeandRecreationalSafety/Falls/index.html

[3] Müller D, Borsi L, Stracke C, Stock S, Stollenwerk B: **Cost-effectiveness of a multifactorial fracture prevention program for elderly people admitted to nursing homes.** *Eur J Health Econ* in press 10.1007/s10198-014-0605-5 24818587

[4] Hamedan A, Maqbali MA (2014). History and physical examination of hip injuries in elderly adults. Orthop Nurs.

[5] González N, Aguirre U, Orive M, Zabala J, García-Gutiérrez S, Las Hayas C, Navarro G, Quintana JM (2014). Health-related quality of life and functionality in elderly men and women before and after a fall-related wrist fracture. Int J Clin Pract.

[6] Gettens S, Fulbrook P: **Fear of falling: association between the Modified Falls Efficacy Scale in-hospital falls and hospital length of stay.** *J Eval Clin Pract* in press 10.1111/jep.12226 25040834

[7] Kiani S, Bayan Zadeh M, Tavallaee M, Hogg RS (2010). The Iranian population is graying: are we ready. Arch Iran Med.

[8] United Nations: **World population ageing: 1950–2050, countries of area: Iran (Islamic Republic of).** http://www.un.org/esa/population/publications/worldageing19502050/pdf/113iran%28.pdf

[9] Chan BK, Marshall LM, Winters KM, Faulkner KA, Schwartz AV, Orwoll ES (2007). Incident fall risk and PA and physical performance among older men. Am J Epidemiol.

[10] Baker MK, Atlantis E, Fiatarone Singh MA (2007). Multi-modal exercise programs for older adults. Age Aging.

[11] Salehi L, Efekhar H, Mohammad K, Taghdisi MH, Shojaeizadeh D (2010). Physical activity among a sample of Iranian’s aged above 60 years: an application of the transtheoretical model. Arch Iran Med.

[12] Vellas B, Wayne S, Romero L, Baumgartner R, Garry P (1997). Fear of fall and restriction of mobility in elderly fallers. Age Ageing.

[13] Keskin K, Borman P, Ersöz M, Kurtaran A, Bodur H, Akyüz M (2008). The risk factors related to fallen elderly females. Geriatr Nurs.

[14] Curcio CL, Gomez F, Reyes-Ortiz CA (2009). Activity restriction related to fear of fall among older people in the Colombian Andes Mountains. J Aging Health.

[15] Freitas SMS, Wieczorek SA, Marchetti PH, Duarte M (2005). Age-related changes in human postural control of prolonged standing. Gait Posture.

[16] Belza B, Walwick J, Shiu-Thornton S, Schwartz S, Taylor M, LoGerfo J (2004). Older adult perspectives on physical activity and exercise: voices from multiple cultures. Prev Chronic Dis.

[17] Wallace KA, Lahiti E (2005). Motivation in later life a psychosocial perspective. Top Geriatr Rehabi.

[18] Hulton L, Frame R, Maggo H, Shiralcawa H, Mulligan H, Waters D, Hale L (2009). The perceptions of PA in an elderly population at risk of fall: a focus group study. NZ J Physiother.

[19] World Health Organization Regional Office for Europe: **What does physical activity mean?** http://www.euro.who.int/en/health-topics/disease-prevention/physical-activity/news/news/2011/02/being-physically-active-helps-prevent-cancer/what-does-physical-activity-mean

[20] Washburn RA, Smith KW, Jette AM, Jannery CA (1993). The physical activity scale for the elderly (PASE): development and evaluation. J Clin Epidemiol.

[21] Marcus BH, Rakowski W, Rossi JS (1992). Assessing motivational readiness and decision making for exercise. Health Psychol.

[22] Ouyang P, Yatsuya H, Toyoshima H, Otsuka R, Wada K, Matsushita K, Ishikawa M, Yuanying L, Hotta Y, Misuhashi H, Muramatsu T, Kasuya N, Tamakashi K (2009). Changes in activities of daily living, physical fitness and depressive symptoms after six month periodic well-rounded exercise programs for older adults living in nursing homes or special nursing facilities. Nagoya J Med Sci.

[23] Janz NK, Becker MH (1984). The Health Belief Model: a decade later. Health Educ Q.

[24] Baert V, Gorus E, Mets T, Geerts C, Bautmans I (2011). Motivators and barriers for physical activity in the oldest old: a systematic review. Ageing Res Rev.

[25] Vaughn S (2009). Factors influencing the participation of middle-aged and older Latin-American women in physical activity: a stroke-prevention behavior. Rehabil Nurs.

[26] Resnick B, Orwig D, D’Adamo C, Yu-Yahiro J, Hawkes W, Shardell M, Golden J, Zimmerman S, Magaziner J (2007). Factors that influence exercise activity among women post hip fracture participating in the exercise plus program. Clin Interv Aging.

[27] Suzuki T (2003). Epidemiology and implications of fall among the elderly. Nippon Ronen Igkkai Zasshi.

[28] Gillespie SM, Friedman SM (2007). Fear of fall in new long-term care enrollees. J Am Med Dir Assoc.

[29] Dergance JM, Calmbach WL, Dhanda R, Miles TP, Hazuda HP, Mouton CP (2003). Barriers to and benefits of leisure time physical activity in the elderly: differences across cultures. J Am Geriatr Soc.

[30] Lee YS (2005). Gender differences in physical activity and walking among older adults. J Women Aging.

[31] Fox KR, Stathi A, McKenna J, Davis MG (2007). Physical activity and mental well-being in older people participating in Better Ageing Project. Eur J Appl Physiol.

[CR32] The pre-publication history for this paper can be accessed here:http://www.biomedcentral.com/1471-2318/14/115/prepub

